# TREM2: Keeping Pace With Immune Checkpoint Inhibitors in Cancer Immunotherapy

**DOI:** 10.3389/fimmu.2021.716710

**Published:** 2021-09-03

**Authors:** Hui Qiu, Zhiying Shao, Xin Wen, Jinghua Jiang, Qinggong Ma, Yan Wang, Long Huang, Xin Ding, Longzhen Zhang

**Affiliations:** ^1^Cancer Institute, Xuzhou Medical University, Xuzhou, China; ^2^Department of Radiation Oncology, Affiliated Hospital of Xuzhou Medical University, Xuzhou, China

**Keywords:** TREM2, immune checkpoint inhibitor, immunosuppressive myeloid cell, cancer immunotherapy, immune microenvironment

## Abstract

To date, immune checkpoint inhibitors have been successively approved and widely used in clinical cancer treatments, however, the overall response rates are very low and almost all cancer patients eventually progressed to drug resistance, this is mainly due to the intricate tumor microenvironment and immune escape mechanisms of cancer cells. One of the main key mechanisms leading to the evasion of immune attack is the presence of the immunosuppressive microenvironment within tumors. Recently, several studies illustrated that triggering receptor expressed on myeloid cells-2 (TREM2), a transmembrane receptor of the immunoglobulin superfamily, was a crucial pathology-induced immune signaling hub, and it played a vital negative role in antitumor immunity, such as inhibiting the proliferation of T cells. Here, we reviewed the recent advances in the study of TREM2, especially focused on its regulation of tumor-related immune signaling pathways and its role as a novel target in cancer immunotherapy.

## Introduction

According to the latest cancer statistics, about 1.9 million new cancer cases and more than 600,000 cancer deaths are projected to occur in the United States in 2021 (1), suggesting that malignant tumor is still a major public health problem worldwide. Over the past decade, tumor patients, especially those with advanced cancers witnessed the mushroom growth of cancer immunotherapies including oncolytic viruses, chimeric antigen receptor T cells, tumor vaccine and immune checkpoint inhibitors (ICIs) (2, 3), among them, ICIs were well on their way to becoming the most promising cancer treatment strategy. To a certain extent, the formerly embarrassing and intractable pattern of cancer therapy has been changed through the use of ICIs, hence Ipilimumab (Yervoy), the first anti-cytotoxic T-lymphocyte-associated protein 4 (CTLA-4) monoclonal antibody (mAb) and Pembrolizumab (Keytruda), the first mAb against programmed death-1 (PD-1) garnered their first global approvals for cancer treatment by United States Food and Drug Administration (FDA) in 2011 and 2014, respectively (4, 5). Soon afterwards, anti-programmed death-ligand1 (PD-L1) mAbs including Atezolizumab (Tecentriq) (6), Avelumab (Bavencio) (7) and Durvalumab (Imfinzi) (8) were successively approved to be used in clinical cancer treatments. These ICIs have remarkably improved the outcomes of some malignancy types, for example, the use of ICIs increased the five-year survival rate of patients with advanced non-small cell lung cancer from 5% to 16%-25% (9). In addition to the above three common ICIs, several novel immune checkpoint targets such as lymphocyte activation gene 3 (LAG3) (10), T cell immunoglobulin and mucin domain 3 (TIM3) (9), T cell immunoglobulin and ITIM domain (TIGIT) (11), V-Domain Immunoglobulin-Containing Suppressor of T Cell Activation (VISTA) (12) and B7-H3 (also known as CD276) (13) were gradually discovered and recognized in recent years, some of them are undergoing clinical trials. However, the overall response rates of ICIs are very low and almost all cancer patients eventually progressed to drug resistance even though the combination therapies (for instance, combining two ICIs (14, 15), adding ICI to chemotherapy (16) or radiotherapy (17) or antiangiogenic therapy (18) were applied to improve the tricky prognosis, this was mainly due to the intricate tumor microenvironment (TME) and complicated immune escape mechanisms of cancer cells.

An increasing number of studies demonstrate that cancer immune evasion is one of the main obstacles in developing satisfactory anticancer therapeutic strategies, and the two key mechanisms leading to the evasion of immune attack are the abnormal expression and activation of immune checkpoints and the excessive formation of suppressive immune microenvironment within tumors (19–21). Hence, inhibition of immune checkpoints alone may not be sufficient to achieve desirable antitumor therapeutic effects, but removing the immunosuppressive microenvironment from tumors probable has a great chance to improve the prognosis of cancer patients and can be used in conjunction with ICIs. In recent years, researchers illustrated that triggering receptor expressed on myeloid cells-2 (TREM2), a transmembrane receptor of the immunoglobulin superfamily, was a crucial pathology-induced immune signaling hub, and more and more evidence suggested that TREM2 played a vital role in tumor-associated macrophages (TAMs) and myeloid-derived suppressor cells (MDSCs). In this study, we first review the structure of TREM2 and the pathway of TREM2 signaling, then we focus on the role and potential of the tumor suppressor TREM2 in the regulation of tumor immune system and cancer immunotherapy.

## TREM2 and TREM2 Signaling Pathway

### Structure of TREM2

TREMs, which were identified as the new activating receptors of immunoglobulin superfamily expressed on human myeloid cells in 2000, include inhibitory and activating isoforms encoded by a gene cluster linked to the major histocompatibility complex (22, 23). Currently, studies had explored several members of TREM family proteins including TREM1 (also known as CD354), TREM2, TREM3, TREM4, plasmacytoid dendritic cell (pDC)-TREM, TREM-like transcript (TLT-1) and TLT-2, among them, TREM2 was an immunosuppressive receptor (24), and it has successfully attracted the attention of oncologists in recent years.

**Figure 1:**
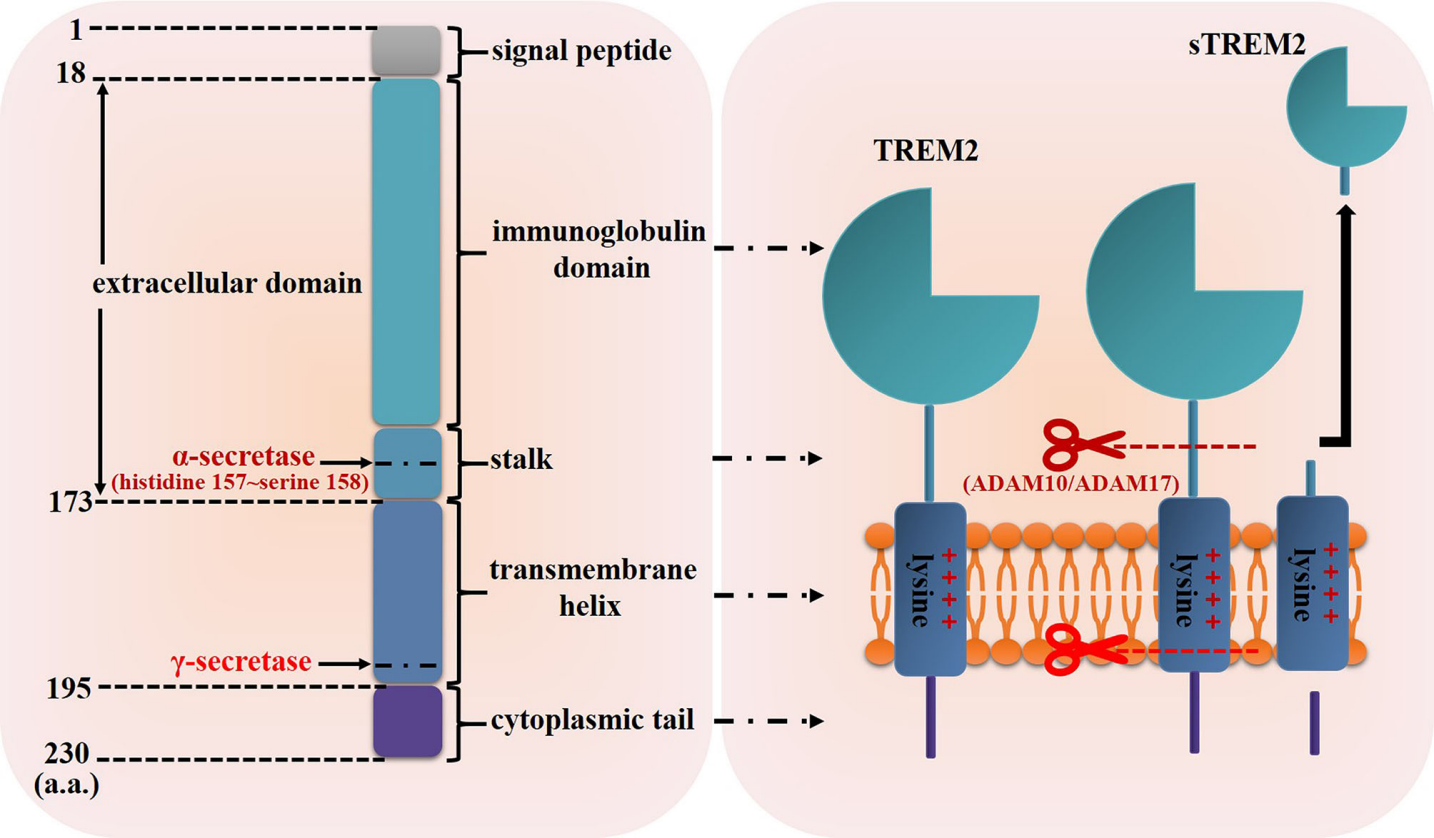
Schematic representation of TREM2 structure. The structure of TREM2 protein mainly includes the signal peptide, extracellular domain which contains an immunoglobulin domain and a short stalk sequence, transmembrane helix and cytoplasmic tail. The α-secretase such as ADAM10 and ADAM17 can cleave TREM2 protein at histidine 157~serine 158 and release sTREM2, then the resultant transmembrane segment undergoes further proteolytic cleavage by γ-secretase.

TREM2 gene, which is located on human chromosome 6p21 with a total length of 4676 base pairs (25), consists of five exons and encodes the glycoprotein TREM2 contains 230 amino acids (a.a.) (**Figure 1**) (22). As a member of single transmembrane hyper-immunoglobulin family, TREM2 was initially cloned as a novel cDNA encoding a TREM1 homologue in 2000 (22). Studies have shown that TREM2 is expressed in some myeloid cells including DCs, monocytes, osteoclasts, Kuppfer cells, alveolar macrophages and microglia (26–32), and its structure is consisted of the following four regions (**Figure 1**) (33, 34) (1): a signal peptide sequence (1~18 a.a.) (2); an extracellular domain (19~173 a.a.): contains an extracellular V-type immunoglobulin domain sequence followed by a short stalk sequence (3); a single transmembrane helix (174~195 a.a.): contains a charged lysine residue (3); a short cytoplasmic tail (196~230 a.a.): lacks signaling motifs.

In addition, TREM2 can be cleaved by sheddases of the metalloprotease family such as a disintegrin and metalloproteases 10 (ADAM10) and ADAM17, and then released as soluble TREM2 (sTREM2) (**Figure 1**) (35, 36). Schlepckow et al. found that both ADAM10 and ADAM17 cleaved TREM2 at histidine 157~serine 158 (37, 38). After being cleaved by ADAMs, the resultant transmembrane segment undergoes further proteolytic cleavage by γ-secretase (35). However, whether TREM2 could be cleaved by other proteases is unknown now. Of greater significance, several studies had detected sTREM2 in the biological fluids (for example, cerebrospinal fluid) of patients suffering from Nasu-Hakola disease, multiple sclerosis and other inflammatory neurological diseases, and the level of sTREM2 was significantly related to disease severity (39, 40), indicating that sTREM2 would not only have a biological function but also a biomarker value.

### TREM2 Signaling Pathway

To dates, studies have shown that TREM2 has several biological functions, including but not limited to cell maturation, cell proliferation, cell survival, phagocytosis and the regulation of inflammation (41–43). This diverse set of functions are mainly regulated by the interaction of TREM2 and a variety of potential TREM2 ligands, which encompass a wide array of anionic molecules including Gram-positive and Gram-negative bacteria (for example, Neisseria gonorrhoeae Escherichia coli and Staphylococcus aureus), DNA, lipoproteins and phospholipids (44–46). Some ligands such as low-density lipoprotein (LDL) and apolipoproteins E (Apo E) are physiologically present in the body, while some ligands including pathological β-amyloid oligomers (Aβ) are released as a consequence of tissue damage and cell death (43).

**Figure 2:**
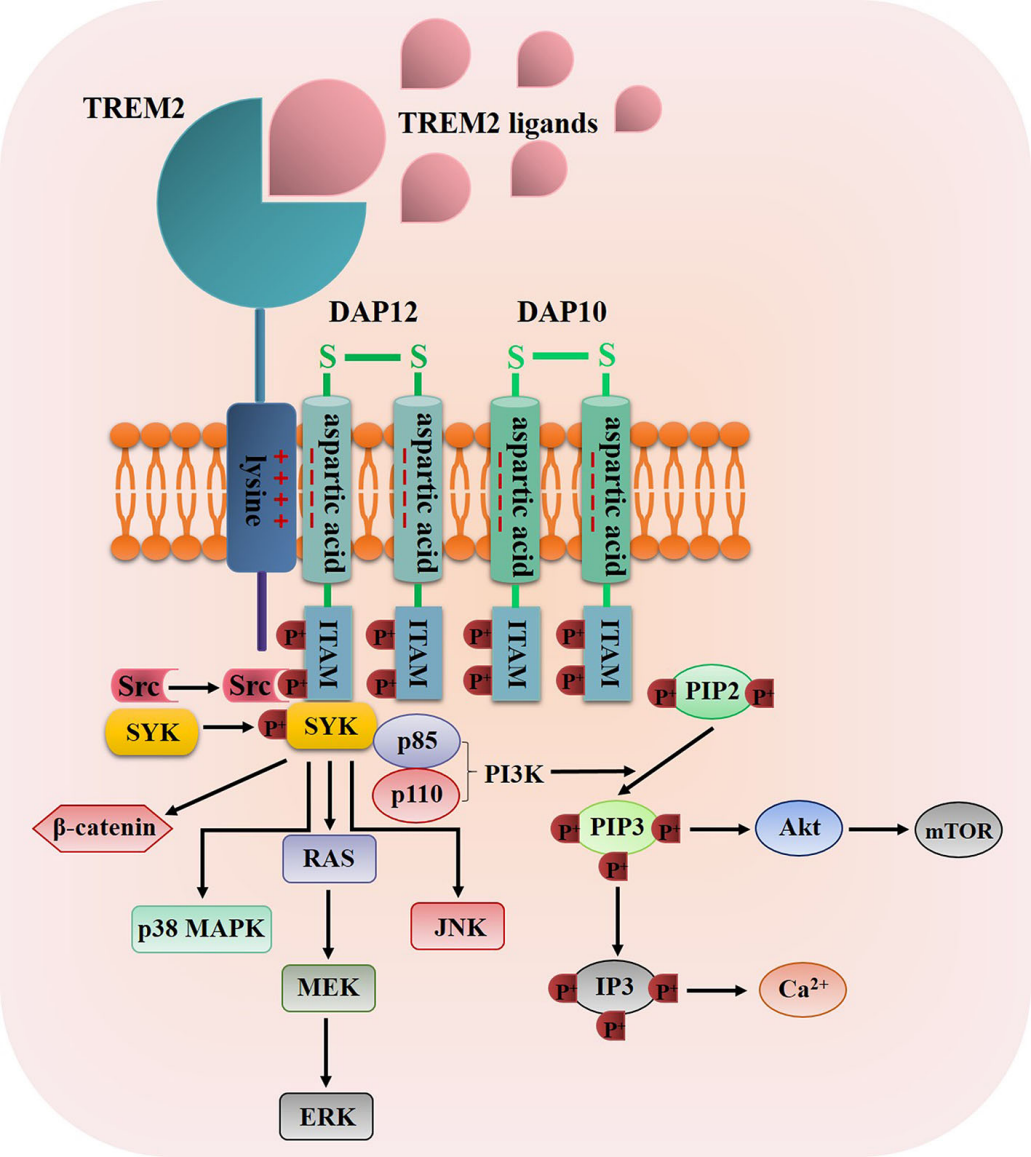
The diagrammatic view of TREM2 signaling pathway. Once TREM2 binding to one of its ligands such as bacterial products, DNA, LDL, Apo E and Aβ, the TREM2 signaling pathway was propagated through the interaction of TREM2 and DAP12/DAP10 *via* oppositely charged residues, thus resulting in the tyrosine phosphorylation of DAP12/DAP10 within its ITAMs by Src tyrosine kinases, then SYK was recruited and phosphorylated by the ITAM regions to activate some downstream signaling molecules such as PI3K, Akt, mTOR, p38 MAPKs, ERK, JNK and β-catenin.

Once TREM2 ligands bind to TREM2, TREM2 will interact with the adaptor proteins DNAX activation protein 12 (DAP12, also known as TYRO protein tyrosine kinase-binding protein) and DAP10 *via* oppositely charged residues, and then the TREM2-DAP12/DAP10 heterodimers are formed (43, 47), for example, the association of TREM2 to DAP12 is induced through a conserved positively-charged lysine in TREM2 (a.a.186) interacts with a negatively-charged aspartic acid residue in DAP12, thus resulting in the tyrosine phosphorylation of DAP12 within its immunoreceptor tyrosine-based activation motifs (ITAMs) by Src tyrosine kinases (48, 49) (**Figure 2**). The main kinase recruited by the ITAM region of DAP12 is spleen tyrosine kinase (SYK), which activates downstream signaling molecules such as phosphatidylinositol 3-kinase (PI3K), serine/threonine protein kinase Akt, mammalian target of rapamycin (mTOR), p38 mitogen-activated protein kinase (MAPK), extracellular signal-regulated kinase (ERK), c-Jun N-terminal kinase (JNK), ultimately leading to cell activation, cell survival and the increase level of intracellular calcium (50–54) (**Figure 2**). Moreover, TREM2-DAP10 also promotes signal transmission by recruiting PI3K and activating Akt and ERK (43, 47). Furthermore, Zheng et al. discovered that TREM2 could stabilize β-catenin by inhibiting its degradation *via* the Akt/GSK3β signaling pathway (55).

Beyond the above signaling pathway, TREM2 also negatively regulates toll-like receptor (TLR) signaling pathways which play crucial roles in the innate immune system by recognizing pathogen-associated molecular patterns (56). Long et al. found that TREM2 could attenuate Aβ1−42−mediated neuroinfammation through downregulating TLR signaling pathway (57). The results of Zhou et al.’s study suggested that lipopolysaccharide (LPS)-induced hyperactive TLR4 might inhibit the negative effect of TREM2 on regulating inflammation, and the imbalance of microglial TLR4/TREM2 might be a potential link between Alzheimer’s disease and systemic inflammation (58). Moreover, the TREM2/TLR4/nuclear factor-kappa B (NF-κB) signaling pathway was illustrated had the function of inhibiting LPS-induced neuroinflammation by regulating microglial M1/M2 polarization (59).

## TREM2: An Emerging Therapeutic Target of Cancer Immunotherapy

### TREM2 Acts As a Tumor Suppressor in Cancer Environment

Researches over the past two decades have illustrated that the immune system could not only inhibit the growth of malignant tumors by destroying cancer cells but also promote the progression of malignancies either by selecting for cancer cells those are better suitable to survive in a host with a strong immune system or by establishing favorable condition within TME that facilitate the proliferation, growth, invasion and metastasis of tumors, this means the immune system has a “double-edged sword” effect on the development of cancers (60).

In order to be fit for cancer cell survival, tumors would coopt myeloid cells, which constitute an important cellular fraction of TME, to suppress the immune system through various mechanisms and then negatively regulate anti-tumor immune function (61, 62). It’s worth noting that recent studies have demonstrated that tumor-infiltrating myeloid cells are heterogeneous and may actually contain both immunostimulatory subpopulation and immunosuppressive subpopulation (63, 64), and MDSCs which can be subdivided as macrophages, granulocytes (especially neutrophils but occasionally and less numerous basophils and mast cells), monocytes and DCs are a immunosuppressive myeloid cell population characterized by the function of promoting tumor growth in tumor immune network (63, 65), this subpopulation also has been reported in relation to the resistance to immunotherapy such as ICIs (66). As some studies revealed, MDSCs possess high levels of arginase I (Arg1) which can induce T-cell anergy by depleting L-arginine, and thus impair T-cell proliferation and cytokine production and inhibit the expression of T-cell receptor CD3ζ chain and antigen-specific T-cell responses (67–69). In addition, the activation of MDSCs lead to the upregulated expression of inducible nitric oxide synthase and the increased production of nitric oxide and reactive oxygen species, and then result in the suppression of antitumor immunoactivity (61). Importantly, MDSCs also obtained the ability of promoting the development and induction of regulatory T (Treg) cells (70, 71). On the other hand, according to several high-dimensional profiling studies, immunostimulatory myeloid cells mainly include type I DCs and M1-like interferon (IFN)-γ-induced macrophages (63, 72, 73), and exert pro-inflammatory and antineoplastic functions through secreting some inflammatory factors (74). Therefore, it is strongly necessary to distinguish immunostimulatory myeloid cells and immunosuppressive myeloid cells, thus helping to deplete immunosuppressive myeloid cells from tumors and induce those cells with immunostimulatory properties to improve the therapeutic effect of anti-cancer immunotherapy strategies.

**Figure 3:**
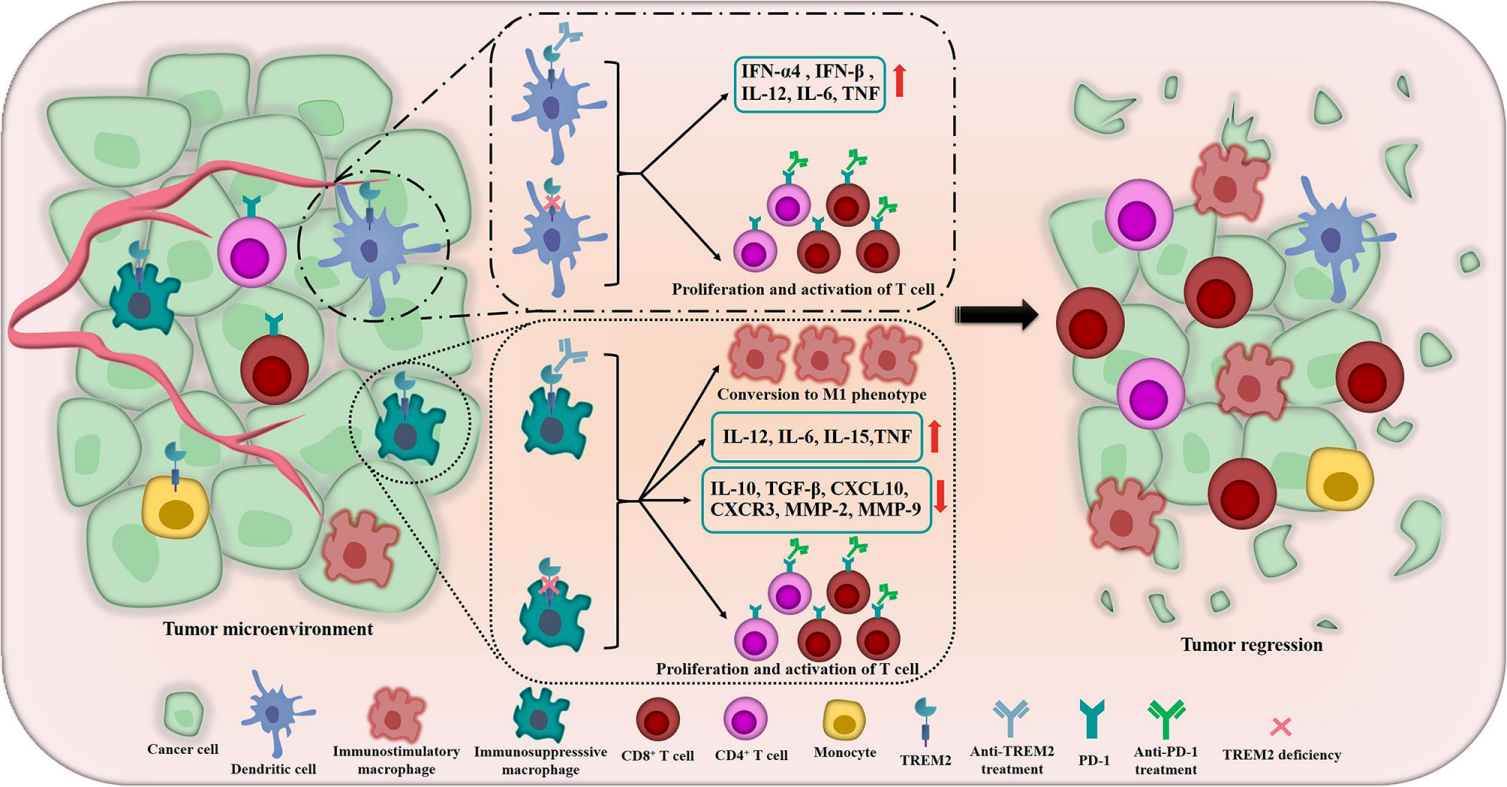
The crucial roles of TREM2 in remodeling TME and inhibiting tumors. In TME, the tumor suppressor TREM2 is highly expressed in some myeloid cells including DCs, immunosuppressive macrophages, monocytes, *etc*. The inhibition of TREM2 could induce DCs produce increased type I IFN (including IFN-α4 and IFN-β), IL-12, IL-6 and TNF. In terms of regulating macrophages, TREM2 deficiency and anti-TREM2 mAb could promote the phenotypic conversion of macrophages to M1 phenotype which have anti-tumor function, meanwhile, the secretion of IL-12, IL-6, IL-15 and TNF are significantly induced and the levels of IL-10, TGF-β, CXCL10, CXCR3, MMP-2 and MMP-9 are decreased. More importantly, TREM2 deficiency and anti-TREM2 mAb also noteworthily promote the proliferation and activation of CD8^+^ T cells and CD4^+^ T cells which expressed PD-1 and potentially improve the responsiveness of cancers to anti-PD-1 treatments. The above crucial roles of TREM2 meaningfully remodel the TME and ultimately promote tumor regression and improve the therapeutic effect of immunotherapy.

Previously, studies had demonstrated that TREM2 acted as a negative regulator of TLR responses in DCs and macrophages (75, 76). TREM2-deficient bone marrow-derived DCs (BMDCs) produced increased type I IFN (IFN-α4 and IFN-β) and inflammatory cytokines including IL-12, IL-6 and tumor necrosis factor (TNF) in response to TLR ligation, furthermore, compared with wild-type BMDCs, TREM2-deficient BMDCs were more efficient at inducing antigen-specific T cell proliferation upon CpG DNA stimulation (76), and Yao et al. got the similar result, they found that TREM2^+^ DCs performed a more potent inhibitory effect on the proliferation of T cells than TREM2^-^ DCs (77) (**Figure 3**). In addition to these, Zhai et al. found that TREM2 had a modulatory effect on the phenotypic conversion of microglia, down-regulation of TREM2 could promote the phenotypic conversion of microglia to M1 phenotype, which induced the secretion of TNF-α, IL-1β and IL-6 and decreased IL-10 and transforming growth factor (TGF)-β (78) (**Figure 3**). Moreover, Wang and his colleagues illustrated that down-regulation of TREM2 significantly decreased the expression of chemokine ligand-10 (CXCL10), chemokine receptor-3 (CXCR3), matrix metalloproteinase-2 (MMP-2) and MMP-9 which played crucial roles in TME (79) (**Figure 3**). These results implied that TREM2 might play a similar role in antitumor immunity. In 2016, Yao et al. elucidated that, compared with normal control, TREM2 was dramatically overexpressed on peripheral blood monocytes and TAMs of patients with lung cancer and tumor-bearing mice, and there was a positive correlation between the level of TREM2 on pulmonary macrophages and the pathological staging or lymph nodes metastasis of lung cancer, the reduction of tumor burden by surgery or chemotherapy induced the remarkable decrease of TREM2 on the peripheral blood monocytes of lung cancer patients (77). In 2020, the research team of Colonna M used single-cell RNA sequencing (scRNA-seq) to dig deep into the tumor-suppressor myeloid cells, they innovatively discovered that TREM2 deficiency and anti-TREM2 mAb treatment could trigger remarkable changes in the macrophage populations infiltrating the tumor, the infiltration of immunosuppresssive macrophages labeled by MRC1 and CX3CR1 was reduced, while the novel subsets expressing immunostimulatory molecules were expanded, moreover, TREM2^–/–^ tumor infiltrates contained more CD8^+^ T cells and CD4^+^ T cells which expressed PD-1 than wild-type infiltrates, suggesting that TREM2 deficiency might significantly promote the activation of T cells and potentially improve the responsiveness of cancers to anti-PD-1 mAbs (73) (**Figure 3**). Then, the authors revealed that TREM2 deficiency could modify the TME in a manner that facilitated partial control the growth of tumor by CD8^+^ T cells (73). At the same time, Amit I’s research team and Weiner A’s research team co-published the parallel excellent and surprising finding, they utilized INs-seq, a new technology for recording scRNA-seq and intracellular protein activity, uncovered a novel Arg1^+^ TREM2^+^ myeloid cells, genetic ablation of TREM2 could significantly inhibit the accumulation of myeloid cells within tumors and lead to immune reactivation (80).

In a word, more and more evidence indicate that TREM2 acts as a tumor suppressor in modulating TME, and the purposeful and accurate regulation of TREM2 signaling pathway has the potential to reshape the TME and exert a stronger anti-tumor effect.

### Emerging and Novel Roles of TREM2 in Cancer Treatment

Up to now, the role of TREM2 in the treatments of malignant tumors is poorly understood, because relevant studies are relatively few, and most of the results are published in recent years. Initially, Wang et al. found that, compared with noncancerous brain tissues, the expression level of TREM2 was significantly increase in glioma tissues, and the overexpression of TREM2 in human gliomas was closely associated with pathological grade and overall survival of patients, furthermore, silencing TREM2 could inhibit the proliferation, migration and invasion of glioma cells (79). Before long, Zhang et al. demonstrated that TREM2 was abnormally upregulated in renal cell cancer tissues, knockdown of TREM2 significantly inhibited the progression of renal cell cancer *via* inactivating PI3K/Akt signaling pathway and increasing the expression of PTEN (81). In 2018, Zhang et al. confirmed that the mRNA and protein expression levels of TREM2 in gastric cancer tissues were significantly higher than those in normal gastric tissues, and the expression level of TREM2 was inversely correlated with the prognosis of patients with gastric cancer (82). In addition, by using a druggable genome small interfering RNA screening library, Duggan et al. found that targeting TREM2 triggered the cell death and reduced tumor burden of esophageal adenocarcinoma (83). These results indicated that TREM2 might be an excellent and effective therapeutic target for cancer treatment.

However, the study of Kim et al. suggested that TREM2 might also acted as a tumor suppressor in some human malignancies, because they detected that TREM2 significantly inhibited the proliferation of colon cancer cells by retarding cell cycle progression and suppressed the tumorigenicity of colon cancer cells through reducing the mRNA expression of pro-tumor cytokines (IL-4) and increasing the mRNA expression of anti-tumor cytokines (IL-12 and IL-15) (84). Importantly, compared with normal colon tissues and human colon carcinoma tissues with stage I, the colon carcinoma tissues with stage II, III and IV expressed lower level of TREM2 protein and this decrease was in a tumor stage-dependent manner (84). More interestingly, even though the expression level of TREM2 was upregulated in human hepatocellular carcinoma tissues, after administrating carcinogen diethylnitrosamine, TREM2^-/-^ mice developed more liver tumors and displayed more deteriorative liver damage, inflammation, oxidative stress and hepatocyte proliferation, and then Esparza-Baquer et al. found that TREM2 played a protective role in the biological process of hepatocarcinogenesis *via* different pleiotropic effects (85). Recently, Wang et al. revealed a novel role of TREM2 in mediating chemoresistance, they found that TREM2 was a potential target of microRNA-149 in gastric cancer, the overexpression of microRNA-149 could decrease the expression of TREM2 and further improve the 5-fluorouracil resistance through β-catenin signaling pathway (86). The above studies show that TREM2 plays different and even opposite roles in the pathogenesis, development and progression of different kind of malignant tumors, and its specific biological functions still need to be further confirmed by a large number of studies.

### Application of TREM2 in Cancer Immunotherapy

Now, a lot of outstanding cancer immunotherapy drugs, especially ICIs, have been successfully transformed from the laboratory to the clinical application, the update and summary on ICIs approved by the FDA is shown in **Table 1** (4, 87, 88). Although ICIs have great promise, only a small population of cancer patients achieve a lasting response to the monotherapy (88). With the use of some predictive biomarkers such as PD-L1 to identify those patients who are more likely to acquire a favorable therapeutic effect from exposure to ICIs, the overall effect of cancer immunotherapy has been improved to a certain extent, but there is still a lot of room for improvement.

**Table 1:** Update and summary of ICIs approved by FDA for cancer treatments.

Immune checkpoints	ICIs	Cancer types or tissue-agnostic conditions (first approved time, year)
**PD-1**	Pembrolizumab (Keytruda)	(1) Melanoma (2014); (2) Non-small cell lung cancer (2015); (3) Head and neck squamous cell carcinoma (2016); (4) Urothelial carcinoma (2017); (5) Classical Hodgkin’s lymphoma (2017); (6) Gastric cancer (2017); (7) Solid tumor with microsatellite instability (MSI-H) or mismatch repair-deficient (dMMR) status (2017); (8) Cervical cancer (2018); (9) Hepatocellular carcinoma (2018); (10) Merkel cell carcinoma (2018); (11) Primary mediastinal large B cell lymphoma (2018); (12) Renal cell cancer (2019); (13) Small cell lung cancer (2019); (14) Esophageal squamous cell carcinoma (2019); (15) Endometrial carcinoma (2019); (16) Bacillus Calmette-Guérin bladder cancer (2020); (17) Colorectal cancer (2020); (18) Cutaneous squamous cell carcinoma (2020); (19) Triple-negative breast cancer (2020); (20) Tumor mutational burden high cancer as determined by an FDA approved test (2020).
**PD-1**	Nivolumab (Opdivo)	(1) Melanoma (2014); (2) Non-small cell lung cancer (2015); (3) Renal cell cancer (2015); (4) Head and neck squamous cell carcinoma (2016); (5) Classical Hodgkin’s lymphoma (2016); (6) Urothelial carcinoma (2017); (7) Solid tumor with MSI-H or dMMR status (2017); (8) Hepatocellular carcinoma (2017); (9) Small cell lung cancer (2018); (10) Esophageal squamous cell carcinoma (2020); (11) Pleural mesothelioma (2020).
	Cemiplimab (Libtayo)	Cutaneous squamous cell carcinoma (2018).
**PD-L1**	Atezolizumab (Tecentriq)	(1) Non-small cell lung cancer (2016); (2) Urothelial carcinoma (2016); (3) Small cell lung cancer (2019); (4) Triple-negative breast cancer (2019); (5) Melanoma (2020); (6) Hepatocellular carcinoma (2020).
**PD-L1**	Durvalumab (Imfinzi)	(1) Urothelial carcinoma (2017); (2) Non-small cell lung cancer (2018); (3) Small cell lung cancer (2020).
	Avelumab (Bavencio).	(1) Urothelial carcinoma (2017); (2) Merkel cell carcinoma (2017); (3) Renal cell cancer (2019).
**CTLA-4**	Ipilimumab (Yervoy)	Melanoma (2011).
	Tremelimumab	Malignant mesothelioma (2015).

In terms of TREM2, the above content has implied that it plays a vital role in immune responses to tumors and is emerging as a novel immunotherapy target. Molgora et al. found that constitutive lack of TREM2 or anti-TREM2 mAb significantly curbed the growth of tumors and led to complete tumor regression when associated with suboptimal PD-1 immunotherapy (73), the encouraging results indicate that therapeutic strategies targeting TREM2 could keep pace with ICIs in cancer immunotherapy. In addition, Xiong et al. re-analyzed a publicly available scRNA-seq dataset of melanoma samples of patients subjected to ICIs and identified a subpopulation of macrophages overexpressing TREM2 that were overrepresented in the non-responding tumors, and this subpopulation of macrophages might contribute to the resistance of ICIs (89). In order to systematically explore the potential immunological functions and the potential prognostic value of TREM2 across 33 cancer types, Cheng et al. conducted a pan-cancer analysis based on datasets from The Cancer Genome Atlas, the Cancer Cell Line Encyclopedia, Genotype Tissue-Expression, cBioPortal and Human Protein Atlas, they used the ESTIMATE algorithm to calculate the immune scores across the 33 types of cancers, their results showed that the expression level of TREM2 was significantly positively correlated with immune scores in diffuse large B-cell lymphoma, acute myeloid leukemia and thymoma, and the levels of immune cell infiltration were significantly correlated with TREM2 expression in most types of malignancies (90), this indicated that TREM2 could function as a prognostic marker in various cancers because of its especial role in tumorigenesis and tumor immunity. Lately, Lee et al. demonstrated for the first time that precursor natural killer (pNK) cells expressed TREM2, compared with wild type mice, the population of pNK cells and the expression levels of NK cell-activating receptors and NK cell-associated genes were all increased in TREM2-overexpressing transgenic mice, on the contrary, the inhibition of TREM2 signaling pathway by TREM2-immunoglobulin or PI3K inhibitor impacted the expression of NK cell receptor repertoire and downregulated the expression levels of NK cell-associated genes, thus significantly impaired the differentiation of NK cells, the results of this study collectively suggested again that TREM2 might act as a novel candidate for cancer immunotherapy (91).

Because of the outstanding function in regulating the immune system, there is currently a clinical trial investigating the effect of a drug named PY314 which targets TREM2 in the treatment of solid malignancies (Clinical Trials No.: NCT04691375), it is a phase 1a/1b open-label study aims to evaluate the safety, tolerability, pharmacokinetics and pharmacodynamics of PY314 as a single agent and in combination with Pembrolizumab in subjects with advanced solid tumors, the drug PY314 was designed to selectively consume immunosuppressive cells and promote the rebalance of TME that facilitates anti-tumor immunity, we expect that satisfactory results would be obtained from this clinical study, and thus further promotes the clinical transformation and application of therapeutic strategies targeting TREM2.

Even though few clinical studies about targeting TREM2 in cancer treatment are conducting now, the potential for clinical translation of TREM2-targeted therapies remains high in the future, and numerous possible interventions are existed to tackle the TREM2 and its signaling pathway (43). The most prominent and direct intervention is to deplete TREM2 genes or target the active domain of TREM2 through using a specific mAb or small molecule which would block the downstream pathways (73). Another potential approach is to target TREM2 ligands, especially condition-specific and/or tissue-specific ligands, but relevant studies is rare now (43). Furthermore, TREM2 is shed by the ADAMs, so the inhibition of the cleaved process is possible to become another feasible intervention (92), but it is noteworthy that the incidence rate of unwanted and inevitable side effects may be high, because ADAM10 and ADAM17 have a wide range of substrates such as junction molecules, adhesion molecules and chemokines and cytokines (93). In order to selectively compete for α-secretase-mediated shedding, Schlepckow et al. identified a mAb named 4D9, which had a stalk region epitope close to the cleavage site, not only stabilized TREM2 on the surface of cells and reducing its proteolytic shedding by ADAMs, but also concomitantly activated the phosphorylation of SYK (94). Finally, the inhibition of the specific intracellular signaling cascade downstream molecules of TREM2 is another different strategy, however, as we all know, DAP12, DAP10, SYK, PI3K, Akt, mTOR and MAPK are also involved in lots of other signal transduction pathways, hence targeting the above molecules may be an ineffective therapeutic method in the context of TREM2-specific treatment.

In conclusion, a growing body of evidence suggests that TREM2 acts as a key signaling hub in tumorigenesis, tumor progression and oncotherapy, and TREM2 may play different or even opposite roles in different malignancies. Therefore, there is still a long and difficult road ahead to explore the exact roles of TREM2 in each malignancy type with different pathologic category and histological origin. More importantly, because TREM2 has been demonstrated by some basic research as an excellent and effective therapeutic target for cancer immunotherapy, more clinical studies or clinical trials are urgently needed to confirm its roles in the treatment of cancer, and promoted the clinical transformation of TREM2 as soon as possible so as to benefit more cancer patients.

## Author Contributions

Conceptualization, LZ and XD. Data curation, HQ, XW, JJ, and LH. Writing—original draft preparation, HQ, ZS, QM, and YW. Writing—review and editing, LZ, HQ, and ZS. Visualization, HQ and XW. Supervision, LZ and XD. All authors contributed to the article and approved the submitted version.

## Funding

This research was funded by National Natural Science Foundation of China, grant number 81972845; Introduction of Specialist Team in Clinical Medicine of Xuzhou, grant number 2019TD003; Graduate Research and Practice Innovation Plan of Jiangsu Province, grant number KYCX21_2660.

## Conflict of Interest

The authors declare that the research was conducted in the absence of any commercial or financial relationships that could be construed as a potential conflict of interest.

## Publisher’s Note

All claims expressed in this article are solely those of the authors and do not necessarily represent those of their affiliated organizations, or those of the publisher, the editors and the reviewers. Any product that may be evaluated in this article, or claim that may be made by its manufacturer, is not guaranteed or endorsed by the publisher.
